# Hard-Rock Stability Analysis for Span Design in Entry-Type Excavations with Learning Classifiers

**DOI:** 10.3390/ma9070531

**Published:** 2016-06-29

**Authors:** Esperanza García-Gonzalo, Zulima Fernández-Muñiz, Paulino José García Nieto, Antonio Bernardo Sánchez, Marta Menéndez Fernández

**Affiliations:** 1 Mathematics Department, Universidad de Oviedo, Oviedo 33007, Spain; zulima@uniovi.es (Z.F.-M.); lato@orion.ciencias.uniovi.es (P.J.G.N.); 2 Department of Mining Technology, Topography and Structures, University of León, León 24071, Spain; antonio.bernardo@unileon.es (A.B.S.); mmenf@unileon.es (M.M.F.)

**Keywords:** hard-rock stability, span design graph, entry-type excavations, support vector machine, extreme learning machine

## Abstract

The mining industry relies heavily on empirical analysis for design and prediction. An empirical design method, called the critical span graph, was developed specifically for rock stability analysis in entry-type excavations, based on an extensive case-history database of cut and fill mining in Canada. This empirical span design chart plots the critical span against rock mass rating for the observed case histories and has been accepted by many mining operations for the initial span design of cut and fill stopes. Different types of analysis have been used to classify the observed cases into stable, potentially unstable and unstable groups. The main purpose of this paper is to present a new method for defining rock stability areas of the critical span graph, which applies machine learning classifiers (support vector machine and extreme learning machine). The results show a reasonable correlation with previous guidelines. These machine learning methods are good tools for developing empirical methods, since they make no assumptions about the regression function. With this software, it is easy to add new field observations to a previous database, improving prediction output with the addition of data that consider the local conditions for each mine.

## 1. Introduction

Entry-type mining methods, such as cut and fill, room and pillar and shrinkage stoping, have been replaced in many mining operations by lower cost, non-entry mining methods, such as sub-level caving. In many mines, however, the nature of the orebody is such that more selective, entry-type mining methods are still desirable. The rock mass stability depends on the state of the stress condition around the openings, the distribution of discontinuities, the strength and the condition of rock mass and, finally, on the dimensions of the stope (the openings made in the process of extracting ore are called stopes or rooms).

The span of the opening (the horizontal distance between the side supports) for a given rock mass condition conforms a single parameter of the design from the many factors that influence the stability of the stopes. There are two limiting constraints that have a decisive influence on the design of the spans between pillars for these excavations [1,2]. Firstly, the nature of entry-type mining is such that workers are exposed to freshly blasted ground. Thus, higher safety factors are required for the design of entry-type room spans than for non-entry excavations. However, as stope excavations are used only for a short time, the high safety factors used for permanent underground civil engineering structures are difficult to justify. Secondly, profitable mining often demands the maximum extraction of ore, which is achieved increasing opening spans between pillars.

Many methods for designing open spans in underground excavations have been developed in the past, often through empirical analysis. The empirical design method called the “critical span graph” was developed by Brennan Lang (University of British Columbia) in 1994 [2] to provide a practical design tool developed specifically for spans in entry-type excavations. It is based on an extensive case-history database of cut and fill mining in Canada, and it defines stable, potentially unstable and unstable rock in span areas on a graph of rock mass rating [3] (RMR${}_{76}$ rock mass performance parameter) against the span between pillars. This graph has been accepted by many mining operations for the initial span design of cut and fill stopes, and it enables an operator to assess the stability of mine openings with respect to a rock mass.

In this paper, a new method for defining stability areas of the critical span graph that applies supervised machine learning classifiers (support vector machine and extreme learning machines) is presented.

The following Section 2, Methods, presents the critical span graph and the problem statement; and it describes the support vector machine and the extreme machine learning methods. Section 3, Results, explains how the methods are applied. In Section 4, the obtained results are discussed. The paper closes with Section 5, Conclusions.

## 2. Methods

### 2.1. Critical Span Graph and Problem Statement

#### 2.1.1. Critical Span Graph

Empirical design methods, which involve the application of knowledge based on documented experience with similar mining conditions, have gained acceptance in the mining industry. This requires a database of observations that relate the stability of the underground structures to mine geometry, the rock mass characteristics and other factors that influence stability [1,2,4]. Empirical methods have been made possible, in part, by widespread acceptance of rock mass classification systems. Empirical design techniques are the only methods available for analyzing the susceptibility of a rock mass to caving due to the characteristics of the process of rock failure. However, empirical methods do not rely on a detailed understanding of failure mechanisms and, as such, are generally only appropriate for preliminary designs [5].

The empirical design methods [1,4,6,7,8,9,10] that have been developed for the design of spans in non-entry stopes and that have gained widespread acceptance in the mining industry would not be suitable for entry-type mining methods, since the definition of stable in a cut and fill stope is much more conservative than in the case of an open stope. Other empirical methods [3,11,12,13,14,15,16] have been proposed as general purpose span design techniques for a range of excavations from temporary mine openings to permanent underground structures. In general, however, they have been derived from databases consisting primarily of civil engineering case histories, which require long-term stability and higher safety factors than those required for entry-type excavation spans.

The empirical design method called the critical span graph was developed by B. Lang [2] at the University of British Columbia, and it was specifically meant for spans in entry-type excavations, by compiling and plotting the 172 observations of a database from entry-type case histories on a span versus RMR${}_{76}$ graph, to enable future prediction of stable spans given the RMR${}_{76}$ of the stope. The span is defined as the diameter of the largest circle that can be drawn within the boundaries of the exposed rock when viewed in plan. Rock mass rating (RMR${}_{76}$) [3] is widely accepted and used as a rock mass classification system. It combines the most significant geomechanical parameters: (1) the strength of intact rock; (2) the percentage of the drill core (RQD); (3) the spacing of discontinuities; (4) groundwater conditions; and (5) the orientation of discontinuities; and it represents them with an overall comprehensive index of rock mass quality. To apply this system, the rock mass is divided into a number of structural domains, and each one is assessed. Weighted ratings were used because the parameters are not equally important. The summation of all parameters yields an RMR value ranging from 0–100. By this approach, we are able to produce a description of the rock mass based on classes defined by its number (for example, RMR<20 is a very poor rock, and RMR>80 is a very good rock). The advantage of this system is that only a few basic parameters relating to the geometry and mechanical conditions of the rock mass are required. Its popularity stemmed from the fact that it could be used for excavation design in rock with significant capacity to predict excavation stand-up time. Because the RMR classification has been updated several times since its initial publications, it is always referred to with a subscript indicating the year (i.e., RMR${}_{year}$) to identify the version of the classification being used. As an example of the application of the RMR${}_{76}$ for the characterization of the rock mass, the mean values of the different geologic parameters and the corresponding values of rock mass quality index are shown in Table 1 in the two main areas of the operation of the Detour Lake Gold Mine [2], where the values of the original database compiled by B. Lang were obtained.

**Figure 1:**
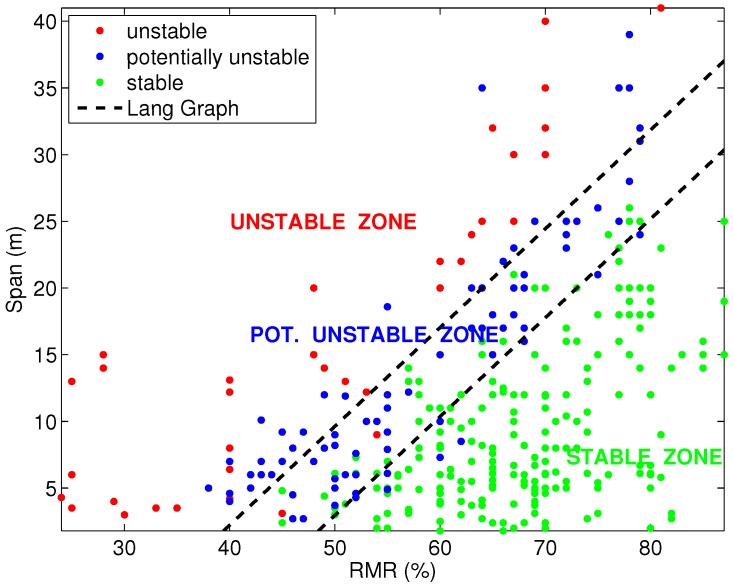
Critical span graph: B. Lang (1994).

The critical span graph developed by Lang consists of two straight lines that divide the RMR${}_{76}$ versus span graph into three zones (stable, potentially unstable and unstable rock), as shown in Figure 1.

In 2002, the database was expanded to 292 observations by a further study conducted by J . Wang [17] at British Columbia University, with the addition of case histories from six more mining operations and using a neural network analysis for the construction of the stability graph, updating the critical span graph.

Subsequently, in 2003, P. Kumar [18] at the British Columbia University incorporated 107 new observations, configuring a final database with 399 cases, updating the critical span graph also using a neural analysis. Although some contradictions in the successive enlargements of the database made by researchers at British Columbia University [2,17,18,19] are observed, the database established in the work of Kumar [18], which is the one that incorporates the largest number of cases, was taken as a reference for conducting the present study. This final database consisted of stope behavior data from eight operating mines in Canada with observational data from 399 operational case histories. The data case history sources are shown in Table 2. Each case history contains information on rock mass conditions expressed as an RMR${}_{76}$ value, span and rock stability.

In this database, the RMR${}_{76}$ ranges from 24–87 and the span from 2–41 m. The RMR${}_{76}$ values for 57% of the cases were concentrated in the 60–80 RMR${}_{76}$ value range. The span values from 3–30 m constitute 95% of the cases. The input data were obtained from different mines that had different personnel surveying the stope dimensions and estimating the RMR${}_{76}$ values. This introduced variability or inaccuracy into the input data. However, the span estimation error should be substantially less than 1 m, which is within the tolerance of the graphical design approach. The variability in estimating the RMR${}_{76}$ value can be more significant and will depend on the level of experience of the engineer conducting the rock mass classification work. For experienced practitioners, the variability for estimating the RMR${}_{76}$ value should be within ±10%. The critical span graph and its updates have been widely accepted in the mining community and provide a quick and simple tool to estimate a maximum span that may be designed based on the observed RMR${}_{76}$ value.

The stability of an excavation (defined in terms of short-term stability because the database is based largely on stoping methods that, by their nature, are of a short duration) is classified into three categories [2]:

1. Stable excavations: (i) no uncontrolled falling of the ground; (ii) no observed movement in the roof; and (iii) no extraordinary support measures implemented.
2. Potentially unstable excavations: (i) extra ground support has been installed to prevent potential falling of the ground; (ii) movement of 1 mm or more in 24 h has been observed in the roof; or (iii) an increase in the frequency of popping and cracking indicating ground movement.
3. Unstable excavations: (i) the area has collapsed; (ii) the depth of failure of the roof is 0.5-times the span (in absence of structure-related failure); or (iii) support was not effective in maintaining stability.

The critical span graph is empirically derived, and therefore, there are some conditions to its application. The users of the graph must always be aware of the limits of the database, which control its applicability [2]. These are: (i) the span is defined as the diameter of the largest circle that can be drawn between pillars and walls in plan view; thus, pillar stability is required for the span design to be reliable; (ii) stopes must have local support installed; (iii) conditions where high stress influences stability cannot be assessed reliably; (iv) the opening roof is horizontal; (v) the term stable refers to short-term stability (approximately three months); (vi) discrete wedges must be adequately supported before this design approach can be used reliably.

#### 2.1.2. Problem Statement

Empirical methods are based on past practice and interpolation among similar input parameters and must be used with a reasonable degree of engineering judgment and adjusted to local conditions. From a mathematical point of view, the empirical methods should be considered as data-driven models. That is, they rely on data to find out specific patterns that can be generalized to a broader range of data.

A mathematical solution to this problem would be a regression functions that would allow one to make predictions for new conditions. In the real world, the regression function is unknown, and data are high-dimensional. However, the classical regression and classification statistical techniques, commonly used for the analysis of historical databases that are based on empirical design methods, stand upon a strict assumption that the underlying probability distribution is known. In contrast, supervised machine learning methods map the input-output relationship from the observed data pairs (input-output) with the hope that this learned mapping would deduce the system response for unknown conditions (unknown input data).

From this perspective, these learning methods can be good tools for developing empirical methods. These learning techniques have the following advantages: they provide a solution for nonlinear and/or unknown systems; they do not need a known distribution to learn from data; they are robust to noise and very effective for sparse, high-dimensional data.

Moreover, the machine learning classifiers analyze input factors assigning a weighting or numerical value to each input factor or factor combination. This is done to provide an estimate or prediction for an output factor. As more information becomes available, the machine learning classifier will adjust and change the input factor weightings to improve output prediction. To the extent that field observations increase, the machine learning methods can optimize the use of that information. As the purpose of a model of this type is the prediction, the procedure of cross-validation is performed, since it allows one to estimate the ability of the model to obtain good predictions for a hypothetical validation set. The goal of cross-validation is testing the model in the training phase, in order to limit problems like overfitting, and giving an insight into how the model will generalize to an independent dataset (i.e., an unknown dataset, for instance, from a real problem).

In this paper, a new method for defining stability areas of the span graph is presented. Two machine learning classifiers are applied: support vector machine (SVM), which is probably the most common method currently used and which offers the advantages of its great adaptability (e.g., to process unbalanced data or probabilistic classification), with generally high prediction accuracy and robustness, but it has the disadvantage of performing a two-class classification (even though a multi-class classification can be obtained from pair-wise classifications); and extreme learning machine (ELM), which offers the advantage of performing multi-class classifications and fast learning speed.

All previous work with the critical span graph [2,17,18] classifies the data into three groups, because field observations are grouped into the categories stable, unstable and potentially unstable. The SVM and ELM classifiers were used following the same classification criteria, but it has also been considered an alternative construction of the critical span graph, which only requires the information of field observations corresponding to the stable and unstable classes, using a probabilistic classification based on SVM that allows one to define soft boundaries between the two classes considered. As these two classes are easier to assess by the engineer, the error due to incorrect assessment is minimized.

Our model represents, thus, an extension with respect to previously available criteria. This model could incorporate additional field observations in a previous database, so that the machine learning classifier continually improves prediction output. This is important because the empirical methods must be used with a reasonable degree of engineering judgment and adjusted according to local conditions. Moreover, since the learning classifiers are multivariate tools, this model could incorporate new control or decision variables that could be registered as field observations.

To extend the applicability of the critical span graph method, it must be developed with a database with samples like those in the local field conditions. Examples of adaptations of the critical span graph to local conditions, which can be perfectly resolved by learning classifiers, are described as follows: (1) T. Brady et al. [1] have investigated the conditions of Nevada gold deposits (USA), which are found in intensely fractured, faulted and argillized host rock (weak rock mass); thirty six case histories from five different mines were added to the original database [2] and used to modify the critical span graph for man-entry mining; and (2) C. Sunwoo et al. [20] have proposed a modified critical span graph that has been successfully used to assess the stability of wide underground openings in six limestone mines in Korea. The database for this modified critical span graph consisted of 140 points from the six mine sites with the RMR values ranging between 40 and 70.

### 2.2. Support Vector Machine Classifiers

The support vector machine (SVM) is an efficient machine learning technique derived from statistical learning theory by Vapnik (1995) [21,22,23] and has proven its good performance in classification, regression [24], time series forecasting and prediction in geotechnical practice and mining science [25,26,27,28,29,30,31]. It can solve non-linear and high-dimensional problems effectively.

A brief introduction about how to construct an SVM model for a classification problem is presented. The main objective of SVM is to find an optimal separating hyperplane that correctly classifies data points and separates the points of two classes as far as possible, by minimizing the risk of misclassifying the training samples and unseen test samples. This means that two classes have maximum distance from the separating hyperplane.

The idea of SVM classifiers can be described as follows: suppose there are *m* observation samples (the training set), $(x_{i},\;y_{i}),\;i=1,2,\ldots ,m$ where:

$$x_{i}^{T}=\left(x_{i1},\ldots x_{id}\right)\in R^{d}$$

is a *d*-dimensional feature of the sample *i* and $y\in \left\{-1,\;+1\right\}$ is its coded class label. If the sample $x_{i}$ is assigned to the positive class, then $y_{i}$ is +1, and if it is assigned to the negative class, then $y_{i}$ is −1.

**Figure 2:**
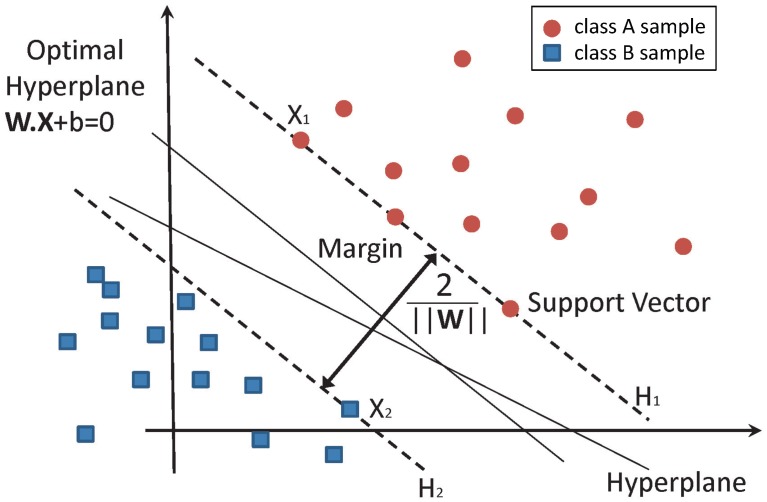
Classification of data by support vector machine (SVM).

This training set can be separated by the hyperplane $w^{T}x_{i}+b=0$, where w is the weight vector and *b* is the bias. The equations of the marginal hyperplanes, $H_{1}$ and $H_{2}$ (Figure 2), are:

$$H_{1}:\;\left(w^{T}x_{i}+b\right)=1$$

and:

$$H_{2}:\;\left(w^{T}x_{i}+b\right)=-1$$

Thus, correctly-classified points satisfy the inequality:

(1) $$y_{i}\left(w^{T}x_{i}+b\right)\geq 1$$

for $x_{i},\;i=1,2,\ldots ,m$.

The distance between marginal hyperplanes (namely, the margin) is equal to $\frac{2}{\left||w|\right|}$. Any training samples that fall on hyperplanes $H_{1}$ or $H_{2}$, the sides defining the margin, are support vectors, as shown in Figure 2.

Thus, the problem is the maximizing of the margin by minimizing $\frac{\left||w|\right|}{2}$ subject to Equation (1). This is a convex quadratic programming problem. Lagrange multipliers $\left(\alpha_{i}>0,\;i=1,\ldots ,m\right)$ are used to solve it:

$$\mathrm{Minimize}\;J_{p}=\frac{{\left||w|\right|}^{2}}{2}+\sum_{i=1}^{m}\alpha_{i}\left[\left(w^{T}x_{i}+b\right)y_{i}-1\right]$$

Calculating the derivative of $J_{p}$ with respect to both w and *b*, the dual problem can be described:

$$\mathrm{Maximize}\;J_{d}\left(\alpha \right)=\sum_{i=1}^{m}\alpha_{i}-\frac{1}{2}\sum_{i=1}^{m}\sum_{j=1}^{m}\alpha_{i}\alpha_{j}y_{i}y_{j}x_{i}^{T}x_{j}$$

$$\mathrm{subject}\;\mathrm{to}\;\sum_{i=1}^{m}\alpha_{i}\cdot y_{i}=0,\;\alpha \geq 0$$

After minimizing $J_{p}$, the optimal weights are:

$$w^{*}=\sum_{i=1}^{m}\alpha_{i}^{*}y_{i}x_{i}$$

where $a_{i}^{*}$ are optimal Lagrange multipliers.

The Lagrange multipliers are non-zero coefficients only for those observations ifor which the constraints are exactly met, that is when $y_{i}\left(w^{{*}^{T}}x_{i}+b\right)=1$. These observations $x_{i}$ are, thus, the support vectors. The optimal bias for any support vector $x_{i}$ is given by $b^{*}=y_{i}-w^{{*}^{T}}x_{i}$.

Thus, the linear decision function can be obtained by the following:

$$f\left(x\right)=\mathrm{sign}\left(\sum_{i=1}^{m}a_{i}^{*}y_{i}x_{i}^{T}x+b^{*}\right)$$

where $a_{i}^{*}$ are the optimal Lagrange multipliers ($a_{i}^{*}$ are non-zero coefficients only for the support vectors $x_{i}$).

In real-world problems, input data are noisy, and no linear separation is possible in the feature space. The hyperplane margin can be relaxed with the introduction of a non-negative slack variable. Hence, the soft margins can be expressed as follows:

(2) $$y_{i}\left(w^{T}x_{i}+b\right)\geq 1-\xi_{i}$$

where $\xi_{i}\geq 0\;\left(i=1,\ldots ,m\right)$ is a slack variable that measures the amount of violation from the constraints.

The optimization criterion to obtain the optimum separating hyperplane should be:

$$\mathrm{Minimize}\;\psi =\frac{1}{2}{\left||w|\right|}^{2}+C\sum_{i=1}^{m}\xi_{i}$$

$$\mathrm{subject}\;\mathrm{to}\;y_{i}\left(w^{T}x_{i}+b\right)\geq 1-\xi_{i},\;\xi_{i}\geq 0\;\left(i=1,\ldots ,m\right)$$

where *C* is a regularization parameter (penalty parameter) that controls the trade-off between maximizing the margin and minimizing the training error.

If the samples are non-linearly separable, the SVM can map the training points, using a function *φ*, to a high-dimensional feature space where linear separation is possible. After a function *φ* is selected, the quadratic programming problem becomes:

$$\mathrm{Maximize}\;J_{d}\left(\alpha \right)=\sum_{i=1}^{m}\alpha_{i}-\frac{1}{2}\sum_{i=1}^{m}\sum_{i=1}^{m}\alpha_{i}\alpha_{j}y_{i}y_{j}K\left(x_{i},x_{j}\right)$$

$$\mathrm{subject}\;\mathrm{to}\;0\leq \alpha_{i}\leq C,\;\sum_{i=1}^{m}\alpha_{i}y_{i}=0,\;i=1,\ldots ,m$$

where:

$$K\left(x_{i},x_{j}\right)=\left[\phi \left(x_{i}\right)\cdot \phi \left(x_{j}\right)\right]$$

The function *K* is called the kernel function.

The decision function is accordingly modified as:

$$f\left(x\right)=\mathrm{sign}\left[\sum_{i=1}^{m}a_{i}^{*}y_{i}K\left(x_{i},x_{}\right)+b^{*}\right]$$

where $a_{i}^{*}$ are optimal Lagrange multipliers ($a_{i}^{*}$ are non-zero coefficients only for the support vectors $x_{i}$).

Two of the kernel functions most commonly used in SVM classifiers are:

$$\mathrm{Linear}\;K\left(x_{i},x_{j}\right)=x_{i}^{T}x_{j}$$

$$\mathrm{Radial}\;\mathrm{Basis}\;\mathrm{Function}\;\left(\mathrm{RBF}\right)\;K\left(x_{i},x_{j}\right)=\exp\left(-\frac{1}{\sigma }\cdot \left|\right|x_{i}-x_{j}{\left|\right|}^{2}\right),\;\sigma >0$$

In the present study, the classification problem is solved using LIBSVM software [32].

Cross-validation will be used to assess the quality of the model and avoid over-fitting [32,33]. It will be applied at two stages: firstly, the tuning of the model parameters (*C* and *σ*) and, then, the validation of the chosen model. The cross-validation process divides the data into a fixed number of equal (or approximately equal) datasets, called folds, randomly chosen. If, for instance, five folds are used, an SVM model is obtained with a training dataset composed of all of the datasets but one, that is 80% of the data, and then, this model is tested with the remaining dataset, that is the remaining 20% of the data. A value of the accuracy is obtained from this test. The process is repeated in such a way that each of the five datasets, in turn, is used as the testing dataset. At the end of this process, five accuracies have been obtained. The mean accuracy is called the five-fold cross-validation accuracy for the model, and it is used to validate the model.

### 2.3. Extreme Learning Machine Classifiers

The extreme learning machine (ELM) algorithm [34] is a generalized single hidden layer feedforward network (SLFN) where the input weights are chosen randomly and the output weights are calculated analytically. For hidden neurons, many activation functions, such as sigmoidal and radial basis functions, can be used, and the output neurons have a linear activation function [35].

Huang [34] showed that the SLFN network, with a sufficient number of hidden neurons, with randomly chosen input weights and hidden bias, can approximate, under the condition that the activation function is infinitely differentiable, any continuous function to any arbitrary level of accuracy. The output weights are determined analytically, so the SLFN network is obtained with very few steps and with low computational cost.

Given a set of *N* observation samples $\left(x_{i},\;y_{i}\right)$, where

$x_{i}=\left[x_{i1},x_{i2},\ldots ,x_{in}\right]\;\in \;R$${}^{n}$ is an n−dimensional feature of the sample *i*,

$y_{i}=\left[y_{i1},y_{i2},\ldots ,y_{im}\right]\;\in \;R$${}^{m}$ is its coded class label.

Then, an SLFN with *L* hidden nodes is modeled as the following sum:

$$\sum_{i=1}^{L}\beta_{i}\cdot g\left(w_{i}x_{j}+b_{i}\right)=\sum_{i=1}^{L}\beta_{i}\cdot h_{i}\left(x_{j}\right),\;j\epsilon \left[1,N\right]$$

where *g* is the activation function, $w_{i}={\left[w_{i1},w_{i2},\ldots ,w_{in}\right]}^{T}$ is the weight vector (input weights) connecting the *i*-th hidden node and the input nodes, $b_{i}$ is the threshold (bias) of the *i*-th hidden node and $\beta_{i}={\left[\beta_{i1},\beta_{i2},\ldots ,\beta_{im}\right]}^{T}$ is the weight vector (output weights) connecting the *i*-th hidden node and the output nodes.

SLFNs can approximate these *N* samples with zero error. In this case, it turns out:

$$\sum_{i=1}^{L}\beta_{i}\cdot g\left(w_{i}x_{j}+b_{i}\right)=y_{j},\;j\epsilon \left[1,N\right]$$

which can be written:

(3) Hβ=Y

where:

$$H={\left(\begin{array}{ccc} g(w_{1}x_{1}+b_{1}) & \cdots & g(w_{L}x_{1}+b_{L})\\ \vdots & \ddots & \vdots \\ g(w_{1}x_{N}+b_{1}) & \cdots & g(w_{L}x_{N}+b_{L}) \end{array}\right)}_{N\times L}$$

$$H={\left(\begin{array}{ccc} h_{1}\left(x_{1}\right) & \cdots & h_{L}\left(x_{1}\right)\\ \vdots & \ddots & \vdots \\ h_{1}\left(x_{N}\right) & \cdots & h_{L}\left(x_{N}\right) \end{array}\right)}_{N\times L}$$

and:

$$\beta ={\left[\beta_{1}^{T}\ldots \beta_{L}^{T}\right]}_{L\times m}^{T},\;Y={\left[y_{1}^{T}\ldots y_{N}^{T}\right]}_{N\times m}^{T}$$

H is called the hidden layer output matrix of the neural network; and the *i*-th column of H is the *i*-th hidden node output with respect to inputs $x_{1},x_{2},\ldots ,x_{N}$.

In the ELM algorithm, for a given number of hidden neurons, it is assumed that the input weights $w_{i}$ and bias $b_{i}$ of hidden neurons are selected randomly, that is they are real numbers. For fixed input weights and biases, the only unknown parameters in SLFNs are the output weights vectors ***β*** between the hidden layer and the output layer, so to train an SLFN is to find a least-squares solution $\hat{\beta }$ of the linear system corresponding to Equation (3).

If the number *L* of hidden nodes is equal to the number *N* of distinct training samples, the hidden layer output matrix H is square and invertible, and SLFN can approximate these training samples with zero error.

However, in most cases, the number of hidden nodes is less than the number of different training samples, so H is a non-square matrix. The smallest norm least-squares solution of the linear system corresponding to the Equation (3) is then:

(4) $$\hat{\beta }=H^{†}Y$$

where $H^{†}$ is the Moore–Penrose generalized inverse of matrix H.

Different methods can be used to calculate the Moore–Penrose generalized inverse of a matrix: orthogonal projection method, singular value decomposition, etc. The orthogonal projection method [36] can be used in two cases: (i) when $H^{T}H$ is non-singular and $H^{†}={\left(H^{T}H\right)}^{-1}H^{T}$; and (ii) when $HH^{T}$ is non-singular and $H^{†}=H^{T}{\left(HH^{T}\right)}^{-1}$.

The special solution $\hat{\beta }=H^{†}Y$ has the following important properties: (i) it is one of the least-squares solutions of Equation (3), which means that it has a minimum training error; and (ii) it has the smallest norm among all of the least-squares solutions of Equation (3). According to Bartlett [37], for feedforward neural networks reaching the smallest training error, the smaller the norms of weights are, the better generalization performance the networks tend to have.

Huang et al. [35] have proposed a version of the ELM algorithm that provides a unified solution for regression and multi-class classification using a kernel matrix (ELM Kernel). From the learning point of view, ELM aims to reach better generalization performance by reaching both the smallest training error and the smallest norm of output weights. Thus, the optimization problem of the objective function for the ELM Kernel can be expressed:

(5) $$\underset{\beta \epsilon R^{L\times m}}{\mathrm{Minimize}:}\;F_{ELM}=\frac{1}{2}{∥\beta ∥}^{2}+\frac{C}{2}\sum_{i=1}^{N}∥\xi_{i}{∥}^{2}=\frac{1}{2}{∥\beta ∥}^{2}+\frac{C}{2}{∥Y-H\beta ∥}^{2}$$

where ∥·∥ denotes the Frobenius norm.

The first term in the objective function is a regularization term that controls the complexity of the learned model and, therefore, its generalization performance. The parameter *C* is a user-specified regularization parameter that trades off the norm of output weights and training errors (as in SVM), and it also improves the generalization performance of the model [38].

The optimal solution $\overset{⋆}{\beta }$ that minimizes Equation (5) can be analytically obtained by setting the gradient of $F_{ELM}$ with respect to ***β*** to zero:

$$F_{ELM_{min}}\Rightarrow \overset{⋆}{\beta }-CH^{T}\left(Y-H\overset{⋆}{\beta }\right)=0$$

According to Huang et al. [35], the closed form solution is:

(6) $$\overset{⋆}{\beta }=H^{T}{\left(\frac{I}{C}+HH^{T}\right)}^{-1}Y$$

where I is the identity matrix of dimension *N* and $HH^{T}=\Omega_{ELM}\;\epsilon R^{N\times N}$ is called ELM kernel matrix.

After obtaining the optimal $\overset{⋆}{\beta }$, the decision score, on test point $x\;\epsilon R^{1\times L}$, is determined by the output function of ELM:

$$f\left(x\right)=h\left(x\right)\overset{⋆}{\beta }=h\left(x\right)H^{T}{\left(\frac{I}{C}+HH^{T}\right)}^{-1}Y$$

(7) $$f\left(x\right)={\left[\begin{array}{c} k(x,x_{1})\\ \vdots \\ k(x,x_{N}) \end{array}\right]}^{T}{\left(\frac{I}{C}+\Omega_{ELM}\right)}^{-1}Y$$

where $\Omega_{\mathrm{ELM}}=HH^{T}$, $\Omega_{ELM_{i,j}}=k\left(x_{i},x_{j}\right)$ and $k\left(x\right)=h\left(x\right)H^{T}\;\epsilon R^{1\times N}$ is the ELM kernel vector for the test point x.

The index corresponding to the highest value of $f\left(x\right)\epsilon R^{m}$ is considered the label of x.

In this specific case, that is similar to SVM, the feature mapping h(x) does not need to be known. Instead, its corresponding kernel k(u,v) can be used. Equation (6) can be analytically solved by a matrix inverse operation, while a constrained quadratic programming problem is required in SVM. This makes the ELM Kernel easy and efficient to implement.

In the present study, the classification problem is solved using the MATLAB codes of ELM with kernels for multi-class classification published by Nanyang Technological University, Singapore [34].

## 3. Calculations and Results

### 3.1. Calculations

#### 3.1.1. Database

The database used for the application for the learning classifiers was published by Kumar [18] and includes 399 samples classified into three groups: stable (S); unstable (U); and potentially unstable (P).

#### 3.1.2. Imbalanced Data

One of the main problems of the data classification to obtain the span stability graph is that the case-history data are clearly imbalanced, with a higher number of stable samples, the relation stable/unstable being three to one. Imbalanced datasets can present a challenge when training a classifier, since the regular learning algorithm has a natural tendency to favor the majority class by assuming a balanced class distribution or equal misclassification cost. Intuitively speaking, with the advantage in quantity, the majority class tends to push the separating boundary towards the minority side to gain a better classification result for itself. Therefore, the standard learning classifiers will tend to predict better the largest class, and SVM and ELM are no exception.

Different strategies have been proposed to improve the efficiency of learning classifiers when using unbalanced data [39,40,41,42]. In general, these strategies have addressed the issue of class imbalance in two different ways. The first way (preprocessing strategies) is to balance the original dataset. The second way (training and post-processing strategies) involves modifying the classifiers in order to adapt them to the datasets.

1. Preprocessing strategies: These are based on sampling techniques that rebalance the dataset in order to get a new training set where the smallest class is better represented. Over-sampling, where new instances are created, can be used.
2. Training strategies: These procedures modify the training incorporating information related with the proportion of number of samples between the classes. For example, to the extent that a learning classifier includes a cost function associated with the misclassification of the samples, a higher weight can be associated with the cost for the misclassification of samples from the smaller classes. This weight can also be related to the risk associated with a misclassification.
3. Post-processing strategies: In general, these procedures are directed towards changing the weight vector of the decision function or of the determination of a new bias or threshold, in order to adjust the boundary decision by the learning classifier, so providing a good margin for separating the smallest class.

In this paper, the strategy was to assume a cost three times higher for the misclassification of samples from the smaller class. Thus, more importance is given to the misclassification of an unstable sample, because this would be an unsafe situation, while the opposite would not.

#### 3.1.3. Classification with Support Vector Machine

Two different strategies have been used to classify the samples with SVM: separation into three classes (unstable, potentially unstable and stable); and binary separation (unstable-stable) with probabilistic prediction.

##### Classification into Three Groups

The SVM classification strategy follows the same criterion (classification into three groups) as previous works [2,17,18,19]. The SVM classifier with the linear kernel has been applied. The linear kernel only considers the penalty parameter *C*.

LIBSVM software [32] uses the approach one-against-one when dealing with multi-classes. A binary classifier is constructed of the two classes. Finally, the sample gofor every pair of classes, and all of the samples are assigned to one es to the class it has been assigned to most of the times. The lines that separate the zones are obtained from the binary separation lines.

The model parameter *C* has been tuned to maximize the five-fold cross-validation accuracy using the grid-search method: the cross-validation accuracy of the models that corresponds to the *C* values of a grid is obtained, and the *C* parameter of the model with the highest cross-validation accuracy is considered the optimum. In this case, the grid was constructed in logarithmic scale with an initial value of $log_{2}C=-6$ and a final value of $log_{2}C=6$, the values of $log_{2}C$ being separated by a step of 0.1. The optimum *C* is $2^{-5.1}=0.0292$, and the corresponding model cross-validation accuracy for five folds is 82%.

##### Binary Probabilistic Classification

As an alternative strategy, a binary separation between unstable-stable classes with probabilistic prediction was performed.

In all previous work [2,17,18], the critical span graph is obtained classifying field observations into three categories. An alternative construction of the critical span graph is a probabilistic SVM classification into two categories. It only requires the information corresponding to the stable and unstable classes. Because these two classes are easier to assess by the engineer, the errors due to this cause are minimized.

To make a probabilistic classification, it is necessary to generate the probabilities associated with each sample. Namely, the output of a classifier should be calibrated using the posterior probability. For that, the model is trained with the data, and then, probabilities are assigned with a logistic sigmoid function, which is set according to the conditional probabilities obtained with the training set [43]. Platt scaling [44] has been used.

When performing a non-probabilistic binary separation (U-S), we do not obtain a strip of separation between the two classes, which should correspond to the in-between cases represented by the class P. However, we can use the logistic function to define soft boundaries between the two classes considered (stable vs. unstable) and, thus, as further discussed in the next section, to obtain the critical span graph.

The linear kernel function was used again. The parameter *C* was tuned using grid-search with the same grid as in the three-group case, and the obtained optimum *C* was again $2^{-5.1}=0.0292$. However, in this case, where there are only two groups, the corresponding model cross-validation accuracy for five folds was 98%.

#### 3.1.4. Classification with Extreme Learning Machine into Three Groups

This classification strategy follows the same approach of previous works [2,17,18,19]. MATLAB codes for ELM with kernels called elm_kernel, published by Nanyang Technological University, Singapore [34], were used. The grid-search method was used for the tuning of the kernel parameters, in this case the RBF kernel, *C* and *σ*. The five-fold cross-validation accuracy was found for pairs of $\left(C,\sigma \right)$ and the parameters related to the highest accuracy model retained. The grid was constructed in logarithmic scale with an initial value of $log_{2}C=-6$ and a final value of $log_{2}C=6$, the values of $log_{2}C$ being separated by a step of 0.1. For the *σ* parameter, the initial and final values were $log_{2}\sigma =0$ and $log_{2}\sigma =6$, the values of $log_{2}\sigma$ being separated by a step of 0.1. The optimum parameter values are C=9.85 and σ=8.57, and they correspond to a cross-validation accuracy for five folds of 88%.

### 3.2. Results

#### 3.2.1. Results of Classification with Support Vector Machine

##### Classification into Three Groups

**Figure 3:**
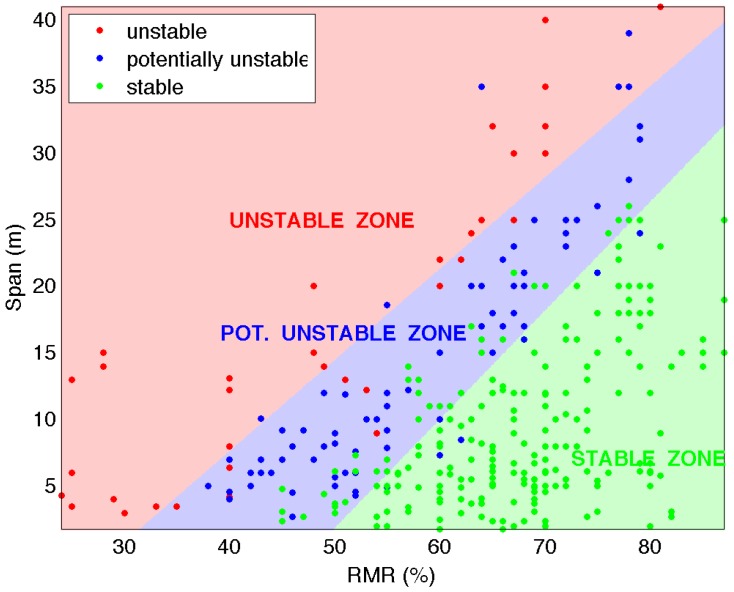
Critical span graph: SVM classification into three groups with the linear kernel.

A linear model to classify the samples into three groups is created with the optimized *C* parameter and the data [18]. Using this model, the points of a grid, with RMR values from 24%–87% and span values from 1.8–41 m and a step of 0.1 for both, are classified and a color assigned (red, unstable; blue, potentially unstable; and green, stable) in the graph. The three different zones limited by straight lines emerge from this classification in the rectangle of study (Figure 3).

It must be taken into account that the span values must be selected, in general under the straight line that allows its classification as the stable class (S). The limit of the stable zone shows that at the lower RMR range, the openings can remain stable only with local support.

##### Binary Probabilistic Classification

**Figure 4:**
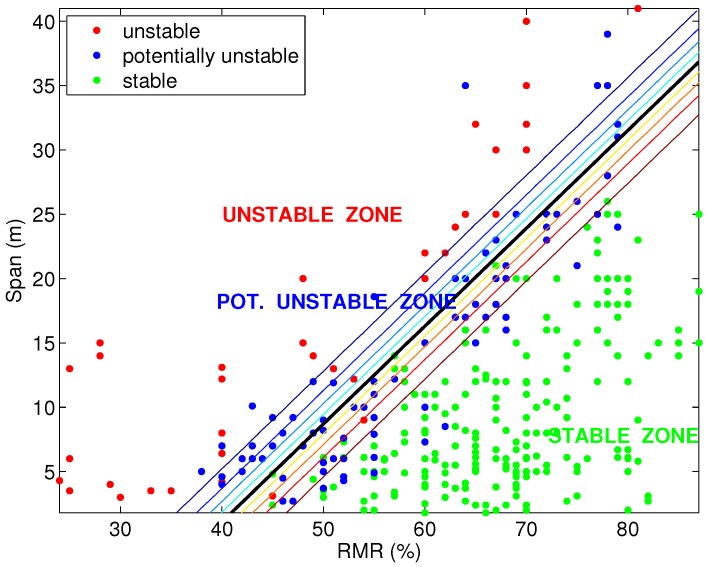
Critical span graph: SVM binary probabilistic classification with the linear kernel.

The result using a binary classification with the linear kernel with probabilistic prediction for the stable and unstable groups is shown in Figure 4. The unstable-stable class separation curve (that corresponds to a stability probability of 50%) has been represented together with the curves that correspond to the probabilities 10%, 20%, 30%, 40%, 60%, 70%, 80% and 90% for the configuration classified as stable.

A strip that separates stable (S) and unstable (U) classes can be obtained using the probabilistic analysis. It can be established assuming that the stable cases (S) correspond to those with a probability higher than 80% of being classified as stable and those with a probability lower than 20%. The strip between these values will correspond to the potentially unstable (P).

#### 3.2.2. Results of Classification with Extreme Learning Machine

**Figure 5:**
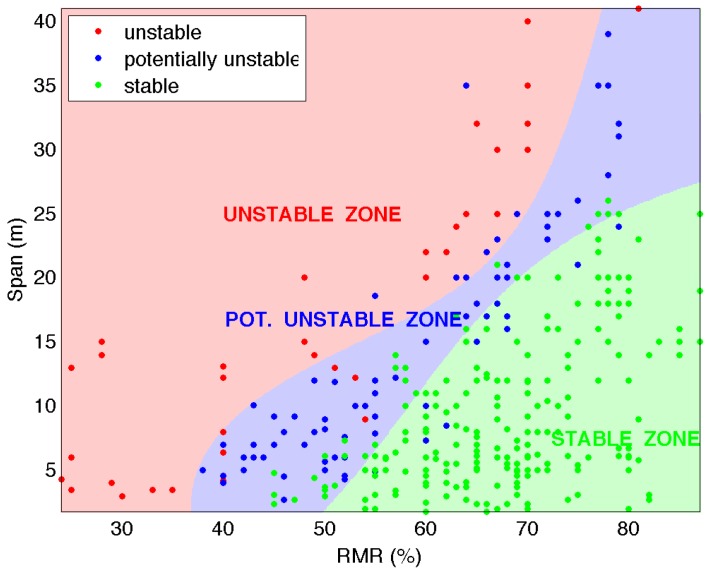
Critical span graph: ELM classification into three groups.

An ELM classifier with the radial basis function (RBF) as the activation function is applied, to classify the points of the same grid of Section 3.2.1 to get a critical span graph, and three zones emerge (stable, potentially unstable and unstable) from the classification of these points, which are shown in Figure 5. It must be taken into account that the span values must be selected, in general, under the line that allows its classification as the stable class (S).

## 4. Discussion

**Figure 6:**
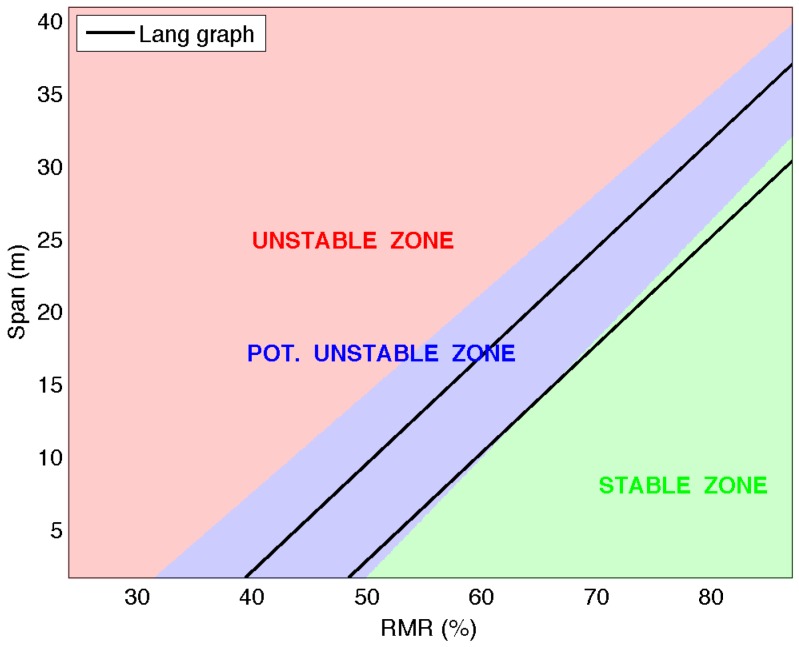
Critical span graph: comparison: SVM (three groups) vs. Lang.

The comparison between the span graph design obtained with SVM for the linear classification into three groups and the Lang critical span graph [2] is presented in Figure 6. Both graphs are very similar, and while the stable region is almost the same, the potentially unstable strip width is larger in the SVM graph. The design criterion obtained with SVM and by Lang [2], in the range of RMR values of the graph and taking into account the limit of the stable zone is practically the same. Although the database published for Kumar (2003) [18] aims to be an extension of the initial database published for Lang (1994) [2], there are some inconsistencies between both databases, which can cause variations in the span graph. Nevertheless, the criterion design for mine-entry excavations obtained in both cases is almost identical.

**Figure 7:**
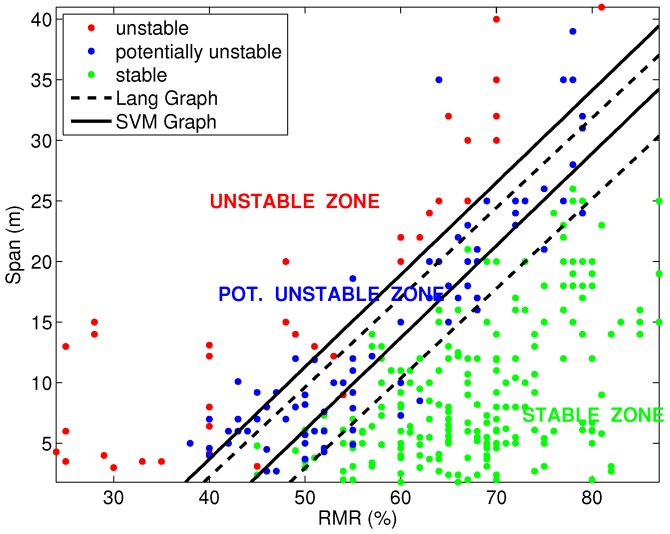
Critical span graph: comparison SVM (probabilistic binary) vs. Lang.

The comparison between the SVM span graph using a probabilistic binary classification (taking as the potentially unstable zone the strip between curves of the 20% and 80% probability of classification as stable) and the initial critical span graph [2] is presented in Figure 7, that shows the Lang graph as dashed lines in contrast to the continuous ones obtained with probabilistic binary classification. It can be observed that the limit of the stable zone proposed by Lang is somewhat more conservative (even though the difference is not significant), while the limit between unstable and potentially unstable zones is very similar to that obtained by Lang.

With this procedure, the probability of having a stable layout being based only on the distribution of stable and unstable span cases of the database was obtained. It can be considered as a qualitative tool for the classification into three groups and to define the critical span graph. However, this probabilistic analysis is not intended to determine the probability of failure, which can be determined only by comparing the number of failed cases to stable ones at a certain probability curve [45,46], and therefore, the probabilistic classification with SVM should not be applied to risk analysis. However, this probabilistic analysis adds an estimation of the sensibility of the forecasted stability to the variations of the quality of the RMR${}_{76}$ and the span value. This could allow a parametric analysis in which a range of possibilities are considered in a conventional deterministic analysis in order to assess the sensitivity of the design.

**Figure 8:**
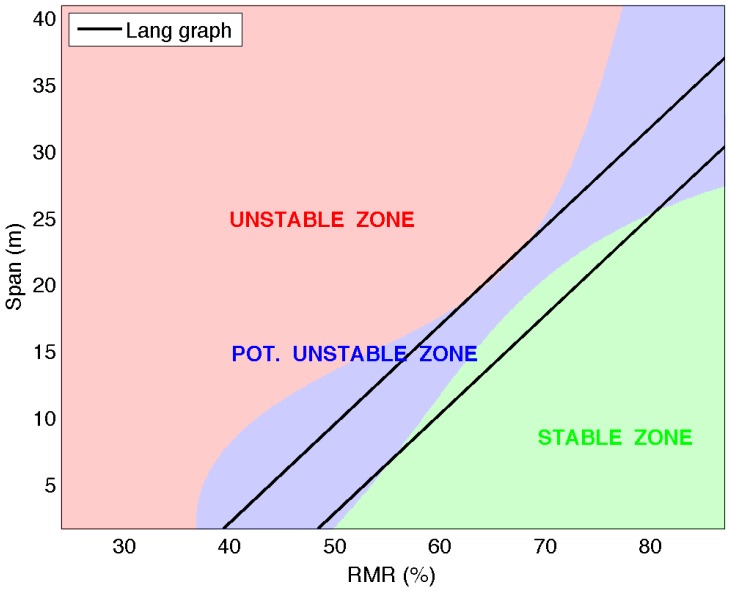
Critical span graph: comparison: SVM (3 groups) vs. Lang.

The comparison between the span graph design obtained with ELM for the classification in three groups and the Lang critical span graph [2] is presented in Figure 8. Both graphics are very similar, and while the stable region is almost the same, the potentially unstable region is slightly wider in the areas corresponding to higher RMR${}_{76}$ and low span in the ELM graph. The design criterion obtained with SVM and by Lang [2], in the range of RMR${}_{76}$ values of the graph and taking into account the limit of the stable zone, is practically the same.

## 5. Conclusions

In this paper, a new procedure for hard-rock stability analysis and determination of the critical span graph for mine-entry excavations, based on learning classifiers (SVM and ELM), is presented.

The proposed critical span graph shows reasonable correlation with previous guidelines that have been accepted by many mining operations for the initial span design of cut and fill stopes. These favorable comparisons suggest that the approach is reasonable for stope design in entry-type mining excavations.

Thus, our model represents an improvement with respect to previously available criteria in the sense that it automatizes the process. This model could incorporate additional field observations in a previous database, so that the machine learning classifier continually improves the prediction output. Moreover, since the learning classifiers are multivariate tools, this model could incorporate new control or decision variables that could be registered as field observations. Furthermore, the cross-validation procedure allows an insight into how the model will generalize to an independent or unknown dataset.

It has also been shown that a classifier with a probabilistic approach can design the critical span graph requiring only the information on the stable and unstable samples. Due to the easier assessment of these kind of samples in mine conditions, this procedure reduces sampling errors.

Since empirical design techniques can only reliably be used in conditions similar to those under which the empirical data were collected, these properties of our model are relevant in order to extend the applicability of the empirical methods to local field conditions. Furthermore, the machine learning software is relatively inexpensive and easy to use, and a mine operator could develop a learning classifier as a successful tool for empirical design.

The critical span graph method can be applied to other field conditions (long-term stability or soft rocks), but the field data should be collected in those specific field conditions for the training of the classifiers. In this case, a critical factor is the correct definition of the behavior considered stable.

## Figures and Tables

**Table 1:** Rock Mass Rating (RMR${}_{76}$) at Detour Lake Mine.

Category	Main Zone		Talc Zone	
	Description	Rating	Description	Rating
Strength	160–180 MPa	13	35–50 MPa	4
RQD	90%	17	80%	16
Joint Spacing	0.4 m	16	0.3 m	9
Joint Condition	smooth, hard, tight	17	smooth surfaces, soft	10
Groundwater	none	10	none	10
Joint Orientation		0		0
Total RMR${}_{76}$		73		49

**Table 2:** Case histories’ data sources [18].

Mines	Cases	Stable (S)	Potentially Unstable (P)	Unstable (U)
Detour Lake Mine	172	94	37	41
Detour Lake Mine	22	10	0	12
Photo Lake Mine	6	0	6	0
Olympias Mine	13	4	1	8
Brunswick Mining	17	5	3	9
Musslewhite Mine	46	35	10	1
Snip Mine	16	12	2	2
Red Lake Mine	107	81	19	7
Summary	399	241	78	80

## Supplementary Materials

The following are available online at www.mdpi.com/1996-1944/9/7/531/s1.
